# Gallbladder Adenocarcinoma With Metastasis in a Young Patient Without Traditional Risk Factors

**DOI:** 10.7759/cureus.103587

**Published:** 2026-02-14

**Authors:** Reem K Busaiba, Alia Lutfi, Leen Jaghel, Ayham Ansari, Abdulsalam Al-Mujammaee, Tarek Alkhouri

**Affiliations:** 1 General Surgery, Thumbay University Hospital, Ajman, ARE; 2 Surgery, Thumbay University Hospital, Ajman, ARE; 3 Medical Oncology, Thumbay University Hospital, Ajman, ARE

**Keywords:** absence of cholelithiasis, chemo-immunotherapy treatment, early-onset gallbladder cancer, gallbladder adenocarcinoma, stage iv gallbladder carcinoma, young adult malignancy

## Abstract

Gallbladder cancer (GBC) is an aggressive malignancy with a poor prognosis, often diagnosed incidentally or at an advanced stage. We describe a case of gallbladder carcinoma in a 30-year-old female presenting with a two-week history of upper abdominal pain radiating to the back, bloating, and excessive gas. Laboratory evaluation showed markedly elevated carcinoembryonic antigen levels, and MRI demonstrated a polypoidal gallbladder fundal mass without evidence of gallstones. The patient underwent laparoscopic cholecystectomy, and histopathology confirmed biliary-type adenocarcinoma and evidence of metastasis. Postoperatively, she was diagnosed with stage IV disease and started on systemic therapy with pembrolizumab, gemcitabine, and cisplatin. After three cycles, PET/CT revealed complete metabolic resolution of metastases, with normalization of tumor markers. This case highlights that GBC can occur in young patients and may present at advanced stages even in the absence of gallstones, emphasizing the importance of early suspicion when evaluating gallbladder masses and the potential effectiveness of combined chemo-immunotherapy in achieving meaningful responses.

## Introduction

With a five-year survival rate of less than 10% in advanced stages, gallbladder carcinoma (GBC) is known for its aggressive nature, poor prognosis, and late presentation. GBC is the most common malignancy of the biliary tract, but it only accounts for 1-2% of gastrointestinal cancers worldwide. The global incidence rate of GBC is approximately 1.2 per 100,000 people, with 122,469 new cases recorded worldwide in 2022 [1]. High prevalence is reported in Northern India, South America, and East Asia, while cases remain relatively uncommon in the Middle East, including the United Arab Emirates (UAE). Furthermore, according to the UAE National Cancer Registry of 2023, there were 56 reported cases of gallbladder and other biliary tract cancers, comprising 16 cases among UAE citizens and 40 among non-citizens. The crude incidence rate per 100,000 is 0.7 for females, 0.4 for males, and 0.5 overall. Despite low incidence, GBC demonstrates a high mortality-to-incidence ratio (~87%), indicating the importance of early diagnosis and surgical intervention [2].

Cholelithiasis is the most significant risk factor, occurring in 85% of GBC patients, with a 10-fold increased risk for larger stones (>3 cm) [3]. Chronic inflammation, recurrent cholecystitis, and the presence of gallbladder polyps, especially large adenomatous types, and genetic predispositions also contribute to malignant transformation. Unfortunately, symptoms are rather nonspecific, mimicking benign gastrointestinal or hepatobiliary conditions. As a result, GBC is often discovered incidentally after cholecystectomy.

This case is remarkably rare for many reasons. The patient presented with GBC without a history of cholelithiasis or family history, suggesting that GBC can occur in young patients outside the expected profile. It also illustrates the diagnostic challenges associated with early-stage detection. Reporting rare cases such as this not only raises awareness of atypical presentations but also supports ongoing research into emerging treatment options, including immunotherapy.

## Case presentation

A 30-year-old female presented to the surgical clinic with upper abdominal pain radiating to the back, associated with bloating and excessive gas for two weeks. The pain was dull, intermittent, and not accompanied by other gastrointestinal symptoms. The patient had a past medical history of diabetes mellitus, hypercholesterolemia, and hypothyroidism, and was currently on medications including metformin, semaglutide, atorvastatin, levothyroxine, vitamin supplementation, and antifungal therapy. Surgical history was notable for keloid removal from the neck and chest wall. She denied any family history of malignant tumors and had no known allergies.

On examination, her vital signs were stable and within normal limits (temperature, 36.5°C; blood pressure, 122/81 mmHg; heart rate, 82 beats/minute; respiratory rate, 18 breaths/minute). Abdominal examination revealed tenderness in the upper abdomen with tympanic percussion and normal bowel sounds. There was no palpable organomegaly, no rebound tenderness, no guarding, and no lymphadenopathy. Examination of other systems, including cardiopulmonary, genitourinary, and musculoskeletal, was unremarkable.

Laboratory investigations showed markedly elevated carcinoembryonic antigen (CEA) at 269.26 ng/mL (normal in nonsmokers: <3 ng/mL, normal in smokers: <5.0 ng/mL) and carbohydrate antigen 19-9 (CA 19-9) at 2.8 U/mL (normal: ≤37 U/mL). Other laboratory findings were unremarkable.

To further evaluate her symptoms, an ultrasound of the abdomen was performed. The gallbladder was well distended. An irregular, heterogeneous echogenic mass with ill-defined margins was identified in the fundal region of the gallbladder, measuring approximately 3.3 × 3.8 cm. A 9.0 mm hyperechoic focus was seen within the mass. An additional, smaller, ill-defined echogenic lesion measuring approximately 1.0 × 1.6 cm was noted near the gallbladder infundibulum. Fine intraluminal sludge was present.

**Figure 1:**
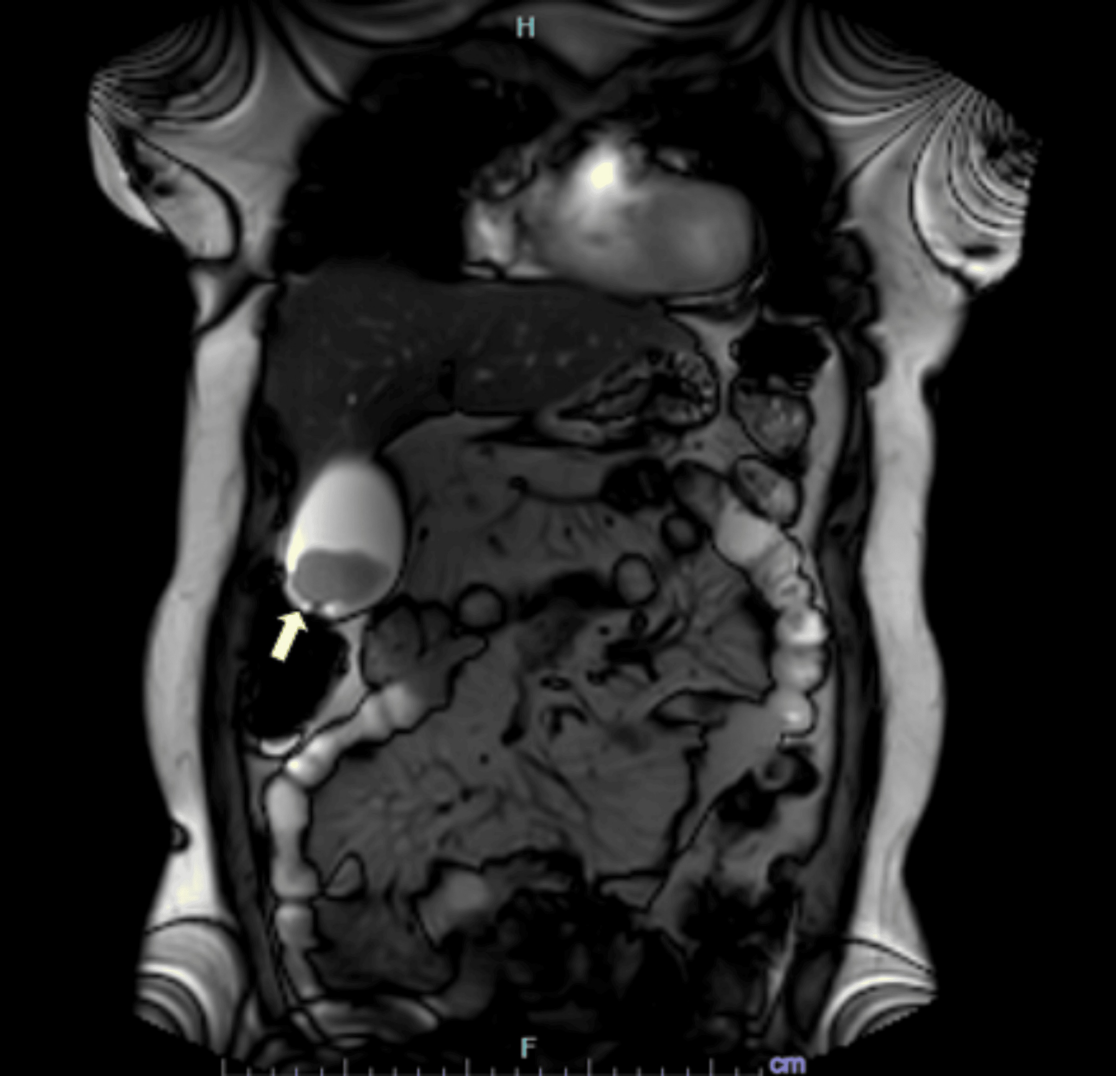
Contrast-enhanced magnetic resonance cholangiopancreatography showing a polypoidal gallbladder fundal mass. Magnetic resonance cholangiopancreatography in coronal view demonstrating a well-defined polypoidal mass arising from the fundus of the gallbladder and projecting into the lumen (yellow arrow).

Furthermore, contrast-enhanced magnetic resonance cholangiopancreatography (MRCP) was performed. The liver was mildly enlarged, measuring 17.2 cm in craniocaudal span, and demonstrated diffuse signal loss on out-of-phase T1-weighted sequences, consistent with mild fatty liver. The gallbladder was well distended, with a well-defined polypoidal mass in the region of the fundus projecting into the lumen, as demonstrated in Figure 1.

**Figure 2:**
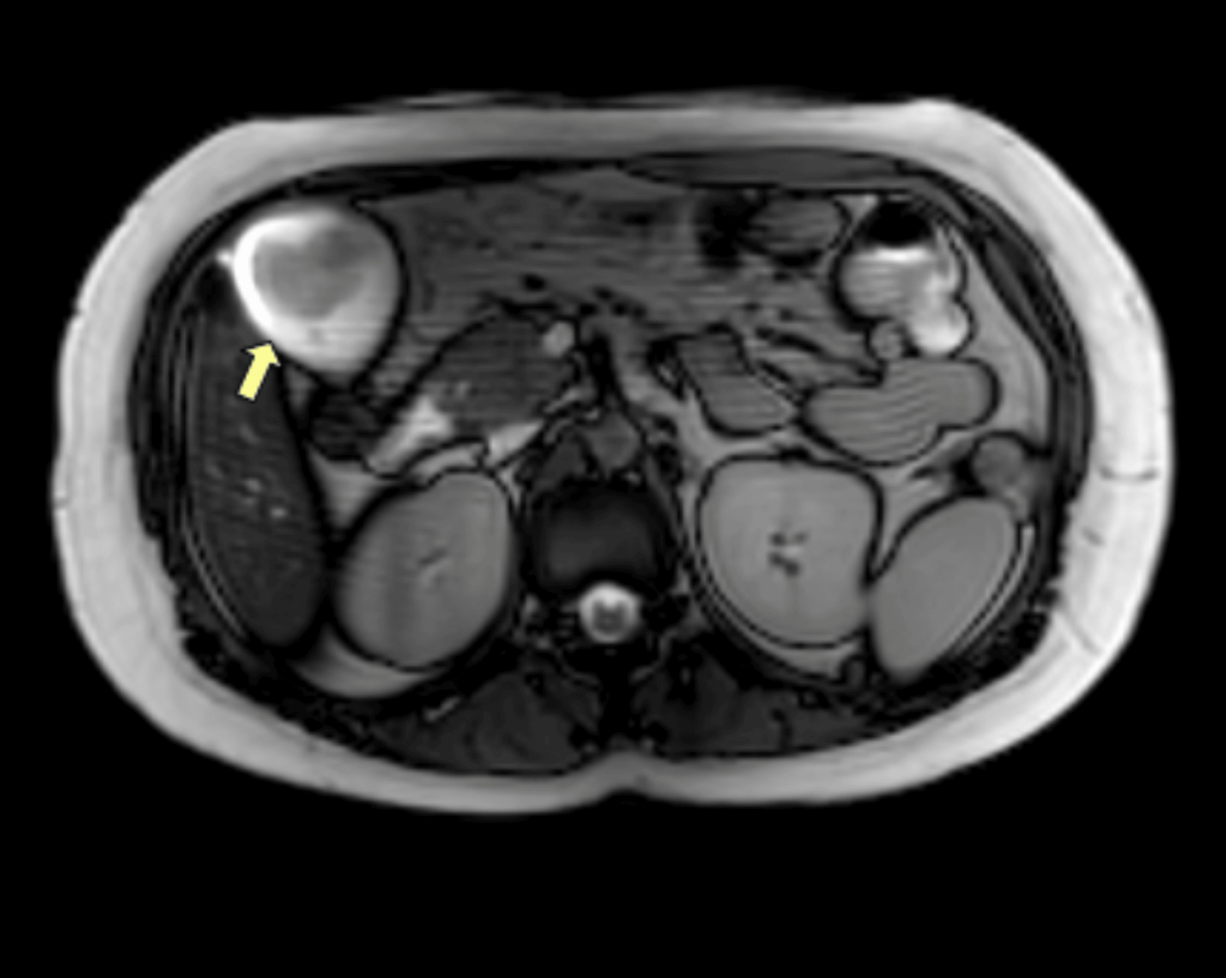
Contrast-enhanced magnetic resonance cholangiopancreatography demonstrating a fundal gallbladder lesion. Magnetic resonance cholangiopancreatography in axial view showing the gallbladder fundal mass with intermediate signal intensity on T1- and T2-weighted sequences, adherent to the gallbladder wall without extension into the pericholecystic planes (yellow arrow).

The lesion displayed intermediate signal intensity on T1- and T2-weighted sequences, was adherent to the gallbladder wall, and measured 4.4 cm × 2.8 cm, without extension into the pericholecystic planes, as demonstrated in Figure 2.

Post-contrast images showed moderate homogeneous enhancement of the mass. Dependent sludge was noted in the lumen, while no signal void suggestive of calculi was observed, and the gallbladder wall thickness was normal.

**Figure 3:**
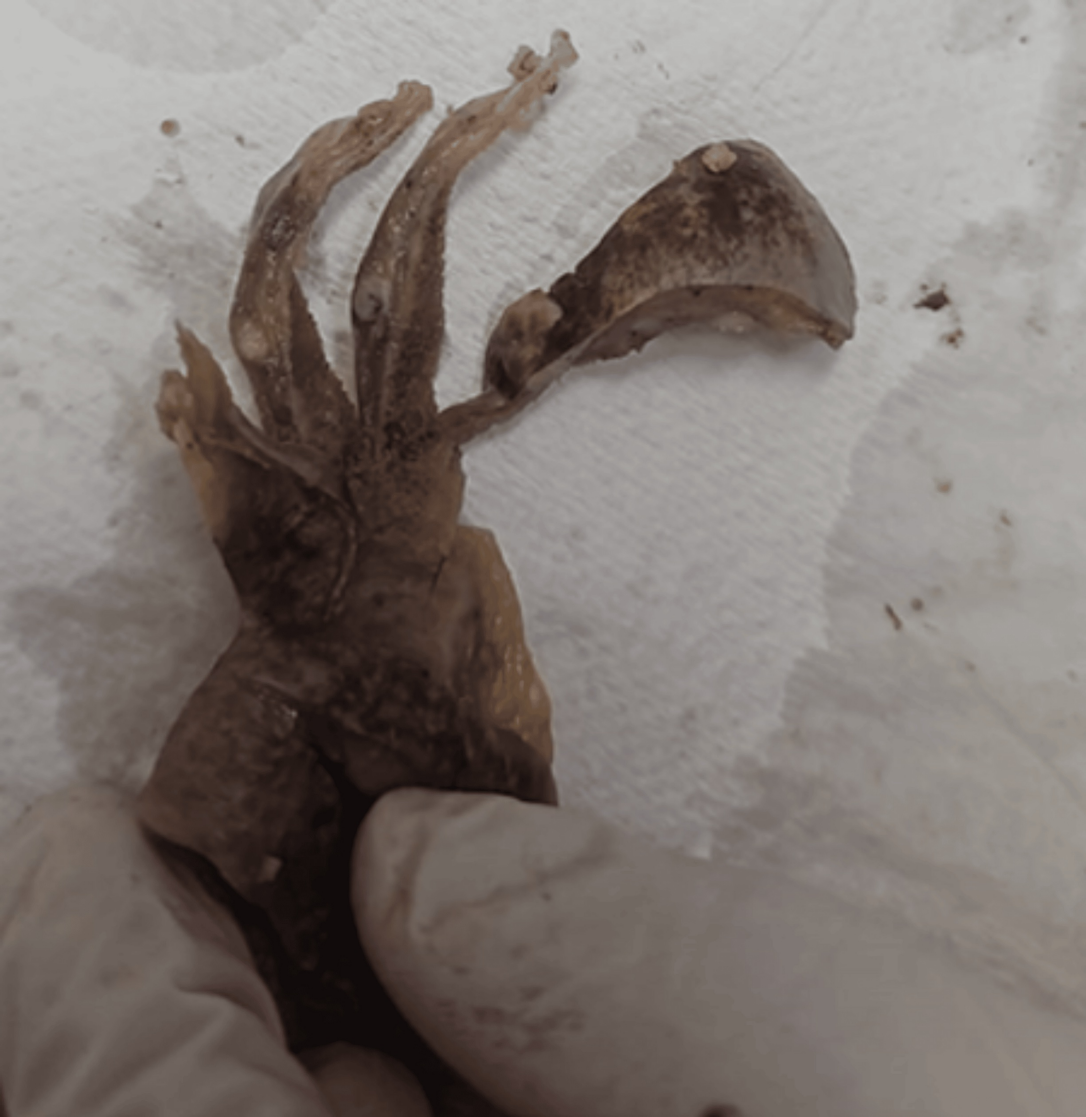
Gross appearance of the resected gallbladder. Gross specimen of the resected gallbladder demonstrating focal puckering at the fundal region with an irregular mass occupying a significant portion of the lumen.

Following the MRCP, the patient underwent a laparoscopic cholecystectomy. The resected gallbladder specimen measured 9.0 × 5.0 × 3.0 cm, with a smooth greyish-white surface and focal puckering at the fundal region, as demonstrated in Figure 3.

On cut section, the lumen was largely occupied by a grey-white, friable, irregular mass attached at the fundus, involving approximately three-quarters of the gallbladder lumen. Serial sections revealed gross extension of the tumor into the liver bed at the serosal surface.

**Figure 4:**
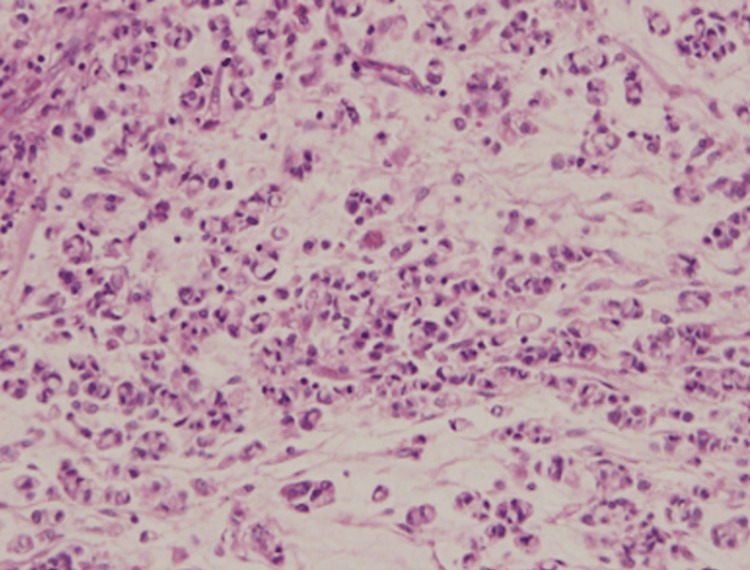
Histopathology of gallbladder adenocarcinoma. Histopathological section of the gallbladder tumor showing biliary-type adenocarcinoma with areas of signet-ring cells and neuroendocrine features (hematoxylin and eosin stain).

Histopathological examination of the tumor, which measured 8.0 × 4.0 × 3.0 cm at the fundal region, revealed a biliary-type adenocarcinoma with areas demonstrating signet-ring cells and neuroendocrine features, as seen in Figure 4.

The final histopathological impression was malignant adenocarcinoma, biliary type, Grade 2, staged as pT3 (tumor perforates the serosa), pM1 (distant metastasis), with pN not assigned.

Additionally, an excisional biopsy of a liver mass located on the right side of the gallbladder fossa was performed. The specimen consisted of a nodular, grey-white soft tissue fragment measuring 5.0 × 1.5 × 2.0 cm. The external surface was inked and trisected. On the cut section, the tissue appeared grey-white to grey-brown, and all tissue was submitted in a single cassette for histopathological evaluation.

**Figure 5:**
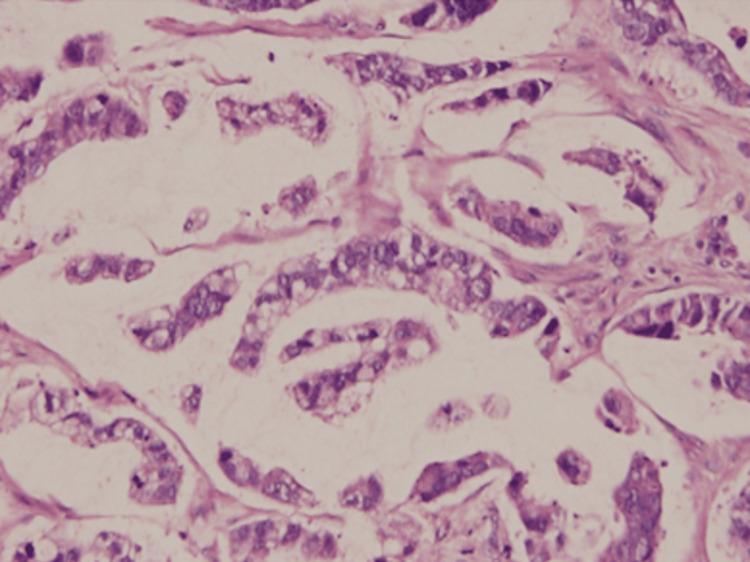
Histopathological evidence of liver metastasis from gallbladder adenocarcinoma. Histopathological examination of the liver biopsy demonstrating metastatic adenocarcinoma with tumor glands containing abundant extracellular mucin and surrounding benign hepatocytes.

Microscopic examination revealed tumor tissue with a peripheral rim of benign hepatocytes. The tumor was composed of glands with abundant extracellular mucin, and the nuclei exhibited marked pleomorphism and coarse chromatin. The peripheral hepatocytes showed aggregates of lymphocytes. These findings were consistent with metastatic adenocarcinoma involving the liver (Figure 5).

Approximately two months postoperatively, the patient was admitted for the first cycle of chemo-immunotherapy due to a diagnosis of stage IV gallbladder adenocarcinoma with liver metastasis. The treatment regimen consisted of pembrolizumab (200 mg in 150 mL normal saline), gemcitabine (1,800 mg in 250 mL normal saline), and cisplatin (150 mg in 1,000 mL normal saline), administered on cycle one, day one.

**Figure 6:**
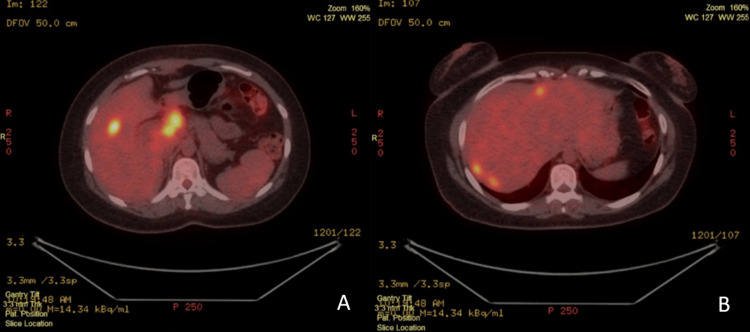
FDG PET/CT demonstrating hepatic and upper abdominal nodal metastases. (A and B) FDG PET/CT scan demonstrating multiple metabolically active metastatic lesions in both hepatic lobes and upper abdominal lymph nodes.

A PET/CT scan was performed, which revealed multiple metabolically active metastatic lesions in both hepatic lobes, as well as metastatic lymph nodes in the upper abdomen (Figure 6).

**Figure 7:**
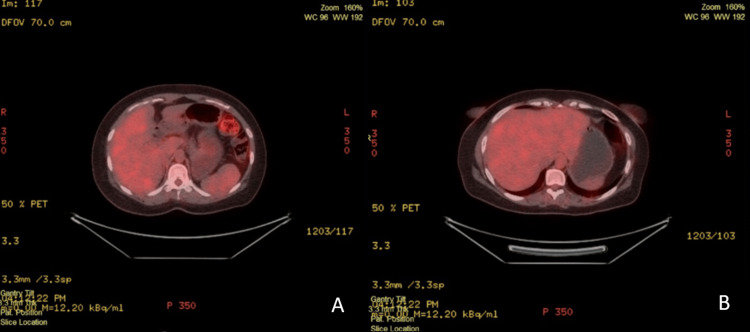
Follow-up FDG PET/CT showing complete metabolic response after chemo-immunotherapy. (A and B) Follow-up FDG PET/CT scan after three cycles of chemo-immunotherapy showing complete metabolic resolution of previously noted hepatic and nodal metastases.

After three cycles of chemotherapy, a follow-up FDG PET/CT study demonstrated a complete metabolic response. This was evident by the interval resolution of previously noted hypermetabolic lesions in both hepatic lobes and the interval resolution of the upper abdominal metastatic lymph nodes (Figure 7).

The patient is responding well to treatment, as reflected in her most recent laboratory results, which show a marked decrease in tumor markers (CEA, 9.58 ng/mL; CA 19-9, <0.60 U/mL). Her diet is being carefully managed and tailored to her clinical needs. Despite the ongoing long course of therapy, she reports significant improvement in her overall well-being, supported by the coordinated care of her surgeons, physicians, nursing staff, and family.

## Discussion

GBC, while relatively rare, remains a highly aggressive malignancy with a high mortality-to-incidence ratio worldwide. The Global Cancer Observatory reported approximately 122,000 cases in 2022, with around 89,000 deaths. The highest incidences are observed in South America and East Asia [1]. Data from the UAE National Cancer Registry (2023) report 56 cases of gallbladder and other biliary tract cancers, including 16 cases in UAE nationals and 40 in non-citizens [2].

Early-stage GBC is often asymptomatic and discovered incidentally during surgery. When symptoms do occur, they are typically nonspecific, including abdominal pain, weakness, nausea, anorexia, weight loss, or jaundice. Advanced disease may present with biliary obstruction or fistulization to adjacent structures [4].

Established risk factors for GBC include female gender, age ≥68 years, chronic inflammation from cholelithiasis, gallstone size ≥1.7 cm, ascending cholangitis, anemia before cholecystectomy, and prolonged duration of cholelithiasis [5]. Chronic inflammation is considered the primary oncogenic driver, though direct injury and exposure to toxins may also contribute. Frequently observed mutations in gallbladder adenocarcinoma include *KRAS*, *TP53*, *CDKN2A*, and *ERBB2* (*HER2*), while hypermethylation of gene promoters is increasingly recognized as a pathogenic mechanism. In some patients, tumorigenesis may follow a pathway similar to other premalignant lesions, such as gallbladder adenomas progressing from dysplasia to invasive carcinoma [6].

Initial laboratory evaluation should include complete blood count, basic chemistry panel, and liver function tests. Right upper quadrant ultrasound is generally the first-line imaging modality, capable of identifying tumors, polyps, gallstones, or biliary obstruction. High suspicion warrants further imaging with CT, MRI, MRCP, or PET/CT [7]. In this case, the management plan prioritized ensuring that no underlying pathology was missed, as demonstrated by prompt evaluation of serum tumor markers and MRCP. While CA 19-9 and CEA are not sufficiently sensitive or specific for diagnosis, they provided useful baseline information and helped guide follow-up, with the patient showing elevated CEA despite normal CA 19-9 [8].

Surgical management of GBC is stage-dependent. T1a tumors are treated with simple cholecystectomy, while T1b tumors may require either simple or extended cholecystectomy. Extended cholecystectomy is generally recommended for T2 or higher, involving wedge resection of the gallbladder bed or segment IVb/V, combined with regional lymphadenectomy. In selected cases, palliative procedures may also be appropriate [9]. In this patient, laparoscopic cholecystectomy served both diagnostic and therapeutic purposes, providing essential histopathological information and relieving presenting symptoms.

Histologically, conventional biliary-type adenocarcinoma is the most common form, while variants such as signet-ring cells or neuroendocrine differentiation are rare and associated with poorer prognosis [10], as observed in our patient.

When appropriate, additional biopsies from suspicious areas, such as adjacent liver tissue, can provide further staging information [11]. In this case, an excisional biopsy of a liver mass in the gallbladder fossa demonstrated metastatic adenocarcinoma with tumor glands containing abundant extracellular mucin and peripheral hepatocytes with lymphocytic aggregates, confirming hepatic involvement. This finding was significant, as it established the presence of metastasis, guided the patient’s staging as stage IV, and directly informed the need for systemic chemo-immunotherapy.

The introduction of combined chemo-immunotherapy has evolved GBC treatment over the past decade. The ABC-02 trial established gemcitabine plus cisplatin as the standard first-line chemotherapy [12], while adding pembrolizumab has demonstrated additional benefit for unresectable or metastatic disease. In this patient, the early and complete metabolic response reflects the cytotoxic effects of gemcitabine and cisplatin, as well as the immune-modulating effects of pembrolizumab [13]. This case emphasizes the importance of early detection, thorough surgical assessment, and combined therapy in achieving favorable outcomes, even in atypical, advanced presentations of GBC outside the traditional risk profile.

The main limitation of this report is that findings from a single patient cannot be generalized. Tumor biology varies widely, and the remarkable response observed may not apply to all advanced GBC cases. While the patient’s molecular profile may have contributed to the outcome, this could not be confirmed due to the absence of genetic testing. Additionally, follow-up was relatively short, preventing conclusions about long-term survival.

Post-treatment surveillance for GBC remains poorly defined and should be individualized. Typically, patients who undergo a cholecystectomy for GBC can be monitored with imaging studies every six months for two years, then annually up to five years. Tumor markers such as CA 19-9 and CEA may be monitored if there are any clinical indications. These recommendations align with the National Comprehensive Cancer Network guidelines for biliary tract malignancies [14].

## Conclusions

GBC can occur in young patients and in the absence of cholelithiasis, underscoring the need for clinicians to maintain a high index of suspicion even outside traditional risk profiles. Early and comprehensive diagnostic evaluation, including tumor markers and advanced imaging modalities such as MRCP and PET/CT, is essential to distinguish malignant from benign gallbladder lesions and to facilitate timely management. Surgical intervention remains both diagnostic and therapeutic, allowing accurate staging and symptom relief while guiding subsequent oncologic treatment decisions.
